# Peer attachment formation by systemic redox regulation with social training after a sensitive period

**DOI:** 10.1038/srep02503

**Published:** 2013-08-26

**Authors:** Mamiko Koshiba, Genta Karino, Aya Senoo, Koki Mimura, Yuka Shirakawa, Yuta Fukushima, Saya Obara, Hitomi Sekihara, Shimpei Ozawa, Kentaro Ikegami, Toyotoshi Ueda, Hideo Yamanouchi, Shun Nakamura

**Affiliations:** 1 National Institute of Neuroscience, NCNP, Tokyo, Japan; 2 Tokyo University of Agriculture and Technology, Life Science and Biotechnology, Tokyo, Japan; 3 Meisei University, School of Science and Engineering, Tokyo, Japan; 4 Saitama Medical University, Saitama, Japan

## Abstract

Attachment formation is the most pivotal factor for humans and animals in the growth and development of social relationships. However, the developmental processes of attachment formation mediated by sensory-motor, emotional, and cognitive integration remain obscure. Here we developed an animal model to understand the types of social interactions that lead to peer-social attachment formation. We found that the social interaction in a sensitive period was essential to stabilise or overwrite the initially imprinted peer affiliation state and that synchronised behaviour with others based on common motivations could be a driver of peer social attachment formation. Furthermore, feeding experience with supplementation of ubiquinol conferred peer social attachment formation even after the sensitive period. Surprisingly, the experience of feeding beyond the cage window was also effective to reduce the required amount ubiquinol, suggesting that peri-personal space modulation may affect socio-emotional cognition and there by lead to attachment formation.

Attachment formation is the most pivotal factor for humans and animals in the growth and development of social relationships^1^,^2^,^3^,^4^,^5^,^6^. Neurobiological studies of social bonding have revealed ubiquitous neural substances involved in attachment formation, including dopamine^7^,^8^, oxytocin^3^, vasopressin^9^, and opioids^10^, which modulate the meso-limbic reward system^9^,^11^,^12^. However, the developmental processes of attachment formation mediated by sensory-motor, emotional, and cognitive integration remain obscure. Social animals, including humans, have a learning mechanism for developing con-specific affiliation, which confers a step-wise acquisition of sensory cognition of con-specific facial feature, chemical signals, calls, and biological motion^13^,^14^,^15^,^16^,^17^,^18^,^19^,^20^,^21^,^22^,^23^. For example, even human neonates that are a few hours old can preferentially respond to a moving object by seeing a facial outline with hair-boundary and they can develop perception of inner facial features like eyes, nose, and mouth within 4 to 5 weeks^13^,^14^. In these studies, the domestic chick (*Gallus gallus domesticus*) was selected as an ideal model since it is a precocial bird (taking food without parenting) and can be experimentally manipulated into not having any sensory experience just after hatching. This allows us to test its naïve affection toward con-specific animals and the effect of its experience on a sensory cue of con-specific animals at a later developmental stage. Social affiliation in chick**s** develops through two well-documented steps: predisposition and imprinting^24^,^25^,^26^. The neuronal substrates of the steps are different and their developments each have a sensitive period^25^,^26^,^27^,^28^,^29^. The former step promotes the latter step^13^,^14^,^15^.

Here we developed a domestic chick model to understand the types of social interactions that lead to peer-social attachment formation and extended the social learning process^28^ beyond the critical periods of predisposition and imprinting. We found that there exists a sensitive period in which social interaction is essential to stabilise or overwrite the initially imprinted peer affiliation state and that synchronised behaviour with other chicks based on common motivations (e.g., food appetite or chasing others out of the pen) could be a driver of peer social attachment formation. Furthermore, food-pecking experience (the party effect) with supplementation of ubiquinol conferred peer social attachment formation even after the sensitive period. Surprisingly, in addition to food pecking within the cage, the experience of food pecking beyond the cage window was also effective, suggesting that peri-personal space modulation may affect socio-emotional cognition and there by lead to attachment formation.

## Result

### Socio-emotional features of grouped and isolated chicks during neonatal development

**Figure 1:**
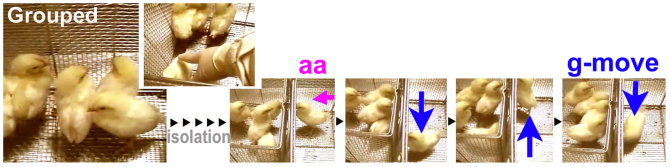
The typical attachment behaviour. A chick temporarily separated from its cage-mates approached the mates (active approach, aa) and went back and forth along the cage fence (grouping movement, g-move).

**Figure 2:**
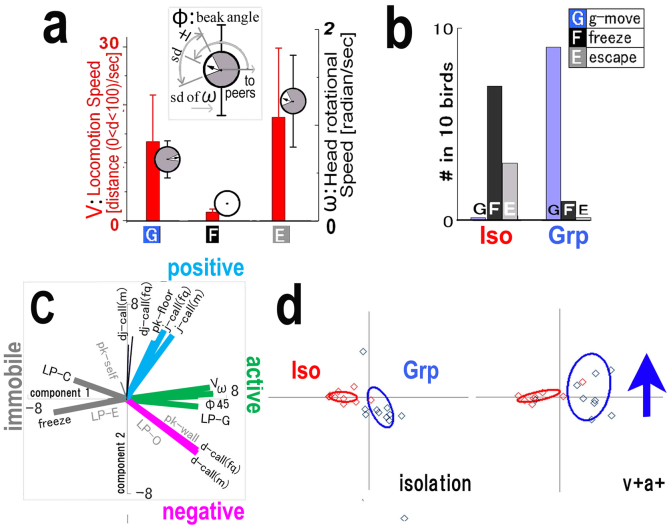
Quantitative comparison of social behaviour in Iso and Grp chicks. (a) Definitions of the behaviour markers: g-move (G), freeze (F), and escape (E). These markers were defined by three parameters—locomotion speed (V, red), head rotation speed (omega, grey), and beak direction (phi, bar angle in grey circle)—based on data pertaining to 79 birds. (b) Social behaviour of isolated (Iso, n = 10) and grouped (Grp, n = 10) chicks were compared with three markers (G, F, and E). (c) The principal component analysis (PCA) of the social test in two contexts. The factor loading vectors with four colour codes representing emotional valence (active; green, immobile; grey, positive; blue, and negative; red). (d) The first and second PCA scores of each animal group are plotted together with the approximated variance ellipse for each rearing condition (Grp or Iso). The blue arrow shows the direction of the Grp ellipse shift from the isolation to v + a+ context.

A domestic chick (*Gallus gallus domesticus*) reared in a group from hatching to two weeks of age developed tight social bonds in the group. When separated from peers temporarily in the next cage, the bird approached the wall bordering the cages and then travelled back and forth along this wall (Figure 1). We referred to this social attachment behaviour as the ‘active approach (aa)' and as ‘g-move' or ‘G' (grouping movement). In contrast, when a chick was socially isolated from the time of hatching (Iso in Figure S1.1b), it froze (F) or escaped (E) upon meeting other chicks (Figure 2b). These typical social behaviours are quantitatively characterised by locomotion velocity (V), head rotation speed (omega), and head horizontal direction (phi = 0; to the peers' cage) such as G and E have high locomotion velocity (V) and head rotation (omega) with opposite beak angle direction (phi), while F has low V and omega value (Figure 2a, Figure S1.1a). The behaviour features were visualised in 3D in time and space (Figure S1.1b) where the 3D coordinate (X, Y, and time) was superimposed with other behavioural factors (see more Figure S1.1). Personal distance was recognised as an important emotional expression^30^,^31^,^32^; thus, each chick's local preference time ratio was examined (Figure S1, 1b2, 4, 6, and 8). The local preference time ratio was the time ratio where the chick stayed in each of the four areas during the specified context tested for two minutes. In the isolation context, the Grp chick explored with a distress call (d-call) (Figure S1.1b5). In contrast, the Iso chick generally froze in the LP-C area (Figure S1.1b2), without any calls (Figure S1.1b1). In the v + a+ context, the behavioural patterns of the two groups were quite different: Grp exhibited the active approach (aa) initially and then g-move (Figure S1.1b7) within the LP-G area (Figure S1.1b8), whereas Iso exhibited freezing (F) (Figure S1.1b3) primarily within the LP-C area (Figure S1.1b4). Furthermore, the behaviour differences of the Grp and Iso chicks were examined by a multi-variate analysis using PCA (Bouquet analysis)^33^,^34^ (Figure 2). We plotted the behaviour features of each bird separately in a PCA plane in each meeting context, and the variance ellipse of each group was shown (Figure 2c, d). Iso and Grp were separated in each context, and Grp changed more between the contexts, which correlated with an increase in active and positive parameter scores (Figure 2d, blue and green). We classified four clusters of factor loading vectors (Figure 2c) in consideration of emotional expression as follows. The active-immobile axis was termed based on movement parameters. The term **active** included head angular velocity (omega), the frequency of head direction towards peers (phi 45), and local preference LP-G, and **immobile** included the freezing behaviour and LP-C. The positive-negative axis was termed primarily based on calls. The term **positive** included J-call, dj-call (intermediate call), and floor pecking, and **negative** consisted of the distress call (d-call)^23^. We recently characterized the chick call behaviour in terms of emotional valence and there exists an consensus about J-call as attracting other chicks to food and d-call as emitted under stressful condition either from physiological or social origin, while dj-call is transitional from d-call to J-call^23^. Pecking behaviour has also emotional valence since the object of pecking suggests the motivational state of chicks, e.g., floor pecking is associated with food pecking behaviour and J-call^23^. These data-driven correlation structures (clusters of behaviour vectors) described the socio-emotional features of each group.

### A sensitive period beyond early imprinting period

**Figure 3:**
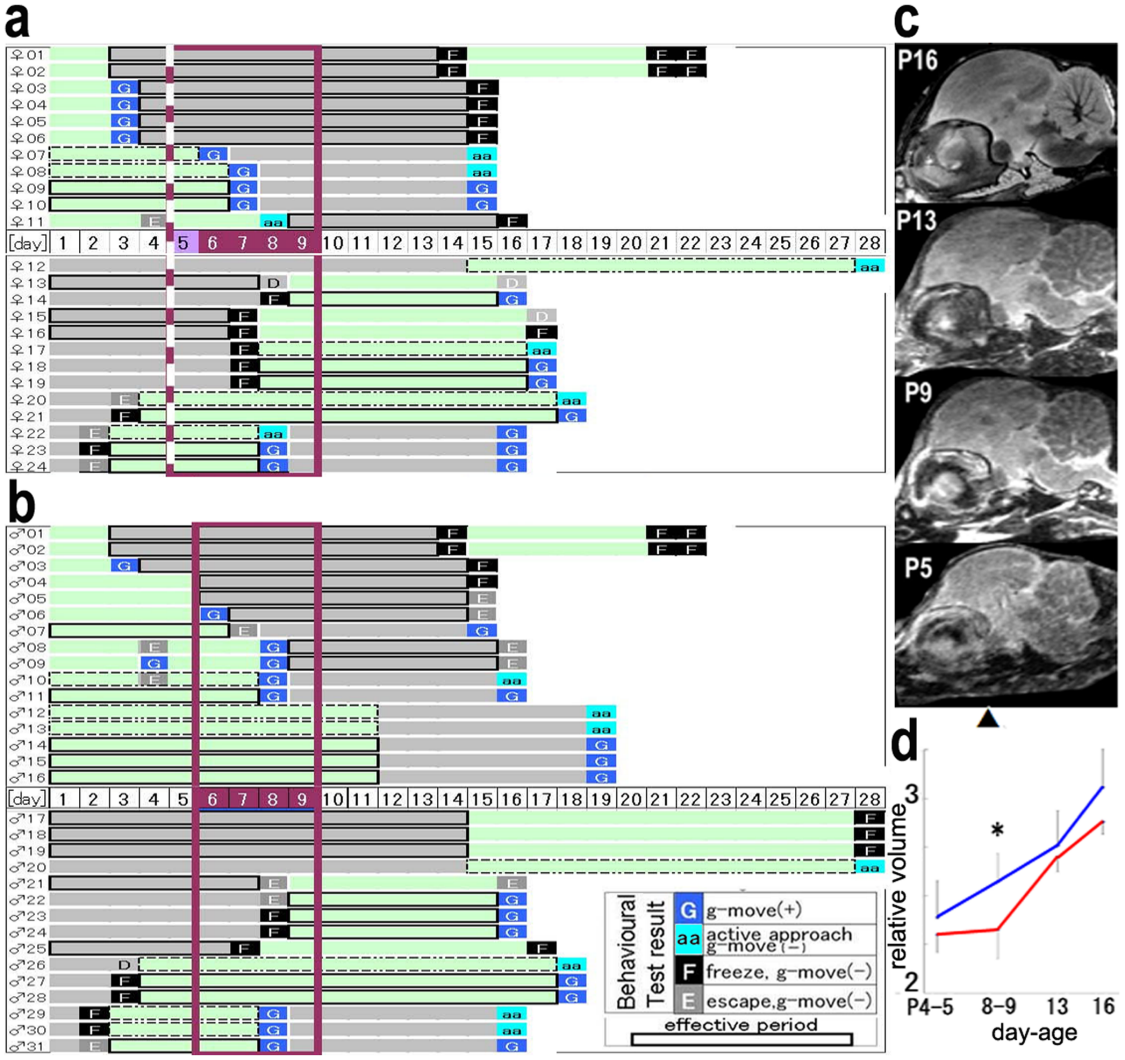
Screening of the sensitive period for the development of social attachment behaviour and brain volumetry. (a, b) Each bar represents the duration of a subject's rearing condition, either grouped (green) or isolated (grey). (a) and (b) were female and male, respectively. A red square demarcates the plausible ‘sensitive' period. (c) Longitudinal 2T-MRI of a representative grouped chick. The sagittal section was aligned along P5 to P16. (d) Significant temporal reduction of forebrain volume of the coronal section around Kuenzel's A8.8–11.0^52^ in Iso chicks in P8–9 (n = 8 each group).

A sensitive (critical) period for imprinting in domestic chicks is well-documented^24^,^25^,^26^,^27^,^28^,^29^, and stress vulnerability develops during early susceptible periods in rodent models^35^. Thus, we examined the existence of a sensitive period of social attachment formation by focusing on four typical behaviour markers (g-move, active approach (aa), freezing, and escape) (Figure 3). We assigned Fifty-five chicks (24 females (a) and 31 males (b)) to one of two rearing conditions. In the first condition, the chicks were first grouped (green bar) and then isolated (grey bar). In the second condition, we reversed the order. The chicks that were initially reared in the Grp condition until postnatal day 6 (P6) maintained their group affiliation even when exposed to the isolated condition from P7 until P14. On the while, the chicks reared in Grp until P2 did not maintain the group affiliation when isolated from P3 until P14. In the reverse condition, the chicks that we reared in Iso until P6 developed social attachment through the grouping condition until P16. However, approximately 50% of the chicks that we reared in isolation until P7 and shifted into group**s** from P8 until P15 subsequently displayed escaping or freezing behaviour. Additional grouping or isolation after P15 did not change the acquired behaviour type until P28. These results suggested the existence of a second sensitive period (P6–P9) for social attachment learning beyond the earlier imprinting period^28^. A longitudinal in vivo study of forebrain volumetric development by T2-enhanced MRI revealed a temporal reduction of the volume around P8–P9 in chicks that we reared in the isolated condition (Figure 3c, d). This result was consistent with the idea of a sensitive period around P6–P9.

### Common motivation as a driver for social affiliation

**Figure 4:**
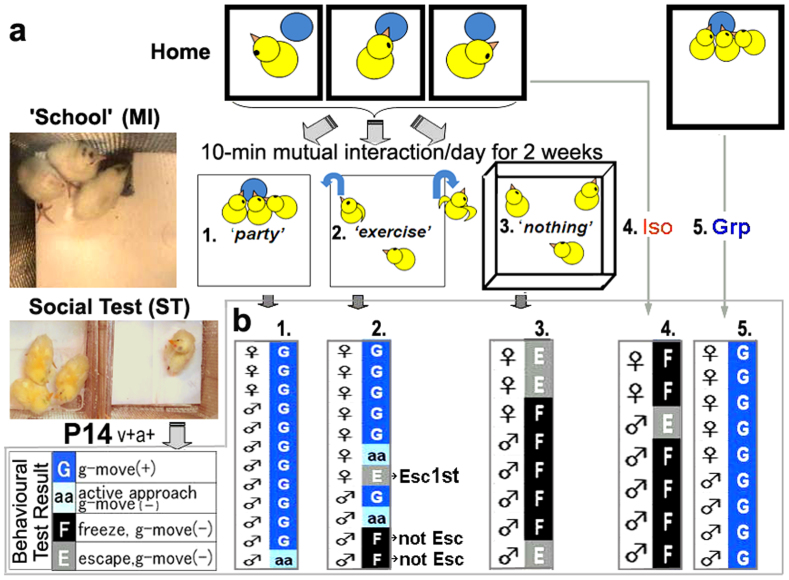
Acquisition of social attachment through the synchronised behaviour driven by a common motivation. (a) Three Iso chicks met one another in the ‘school' cage as a mutual interaction (MI) on postnatal days 1–13 for 10 minutes per day with feeding (1), flying out of the cage (2), or neither (3). (b) At P14, the social test was performed. The results are summarised in the bottom columns. G, aa, E, and F designated g-move, active approach, escape, and freeze, respectively.

Next, we studied how chicks develop social attachment through sensory-motor mutual interaction (MI). For this purpose, we placed three Iso birds in the same cage with food for 10 minutes (school (MI)) per day during P1–P13 (Figure 4). Surprisingly, the chicks that had experienced MI exhibited the social attachment behaviour g-move on the social test (ST) of day 14 (Figure 4b), and the MI with the common appetite motivation was labelled as the ‘party effect'. As another motivation, we introduced a type of social play^6^ we termed as ‘exercise'. We placed Iso chicks in a cage where they could freely jump in and out over the cage walls (Figure 4a2). After one chick jumped out first, other chicks followed this behaviour, but some chicks remained in the cage. Interestingly, the chicks that followed the escaping behaviour of the others expressed the g-move attachment on the social test of P14. In contrast, the chicks that escaped first (Figure 4b2, ‘Esc1^st^') or remained in the cage (Figure 4b2, ‘not Esc') did not develop the g-move. As the control with ‘no motivation', we placed Iso chicks in the same cage but without the possibility of either food or escape (Figure 4a3). These subjects remained in the cage, interacted little with one another, and failed to develop the g-move attachment at ST (Figure 4b3). These results suggested important elements of social skill training that induces mutual interaction and behavioural synchronisation driven by the common motivation in peers to develop social attachment.

### Oxidation-reduction regulation affected affiliation development

**Figure 5:**
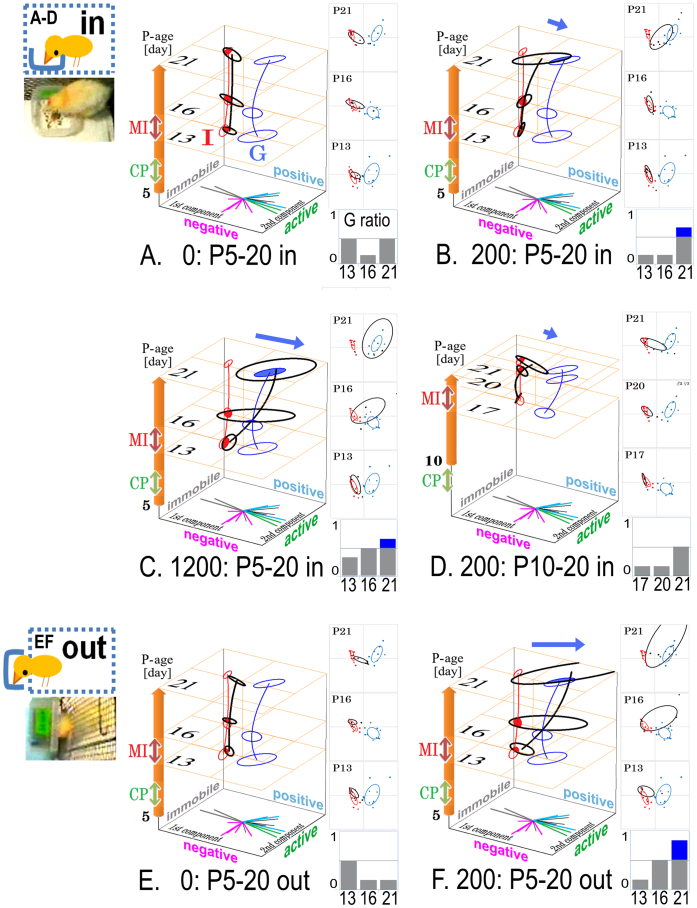
A synergistic effect on social attachment formation with mutual interaction and orthomolecular supplementation of ubiquinol. Attachment formation with MI and ubiquinol. Developmental trajectory was visualised by PCA in 3D time space as the stack of 2D planes at three stages: P13, P16, and P21. The trajectories of each experimental group were colour-coded (Iso in red, Grp in blue, and ubiquinol in black). Ubiquinol was ingested at 0 (vehicle, A, E), 200 (B, D, F), or 1200 (C) [mg/kg weight·day] within the cage (in: A–D) or outside (out: E, F). The behaviour similarity of ubiquinol group with Grp was evaluated by a G ratio based on the Mahalanobis distance (significance was marked with blue bar). Three female and 3 male subjects were used for each condition A–F. Two feeding conditions were set: condition **in**, within the cage (A–D), or condition **out**, taking food through a window of the cage (E, F).

**Figure 6:**
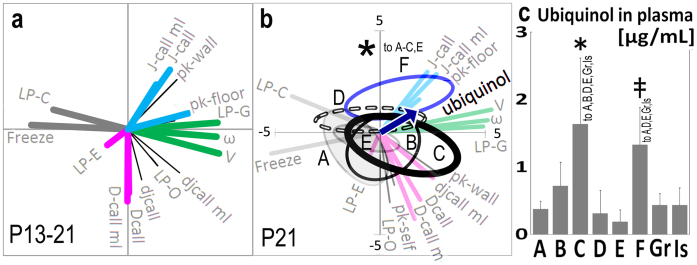
The comparison of acquired peer-social behaviour patterns together with plasma ubiquinol. The behaviour features of various conditions were compared by PCA including serum ubiquinol level. (a) The factor loading vectors of the behaviours of three stages of development. (b) The behaviour data of each condition was compared using the variance ellipses based on PCA of behaviour data and plasma ubiquinol at P21 (blue vector). The distribution of Group F data was significantly different from that of condition A–D and shifted to positive affection (j-call parameter) according to Wilks' lambda analysis (asterisk). (c) Ubiquinol concentration in blood plasma. The significant difference of plasma ubiquinol was observed under two conditions, ubiquinol at high (1200 mg/kg weight·day)with feeding inside of the cage and low (200 mg/kg weight·day) with feeding out (F) by one-way ANOVA and Tukey's post-hoc tests in C (*) and F (double cross).

Finally, we explored treatment models after the sensitive period of peer social learning with the hope that an integrated approach to multi-level treatment would yield a synergistic effect on attachment development. In addition to social skill training (school, MI scheme in this study) based on a common motivation (in this study, food appetite, for example), we introduced an orthomolecular nutritional treatment^36^, which is preferable to psychotic drugs at the conventional dose, especially in a developing child. Among various nutrients, ubiquinol (reduced form of coenzyme Q10) is promising because it functions as a co-enzyme of oxidative phosphorylation, an anti-oxidant^37^, and a neuronal cell protectant^38^. We mixed the daily oral intake of ubiquinol in the food from P5, which is immediately after the beginning of the sensitive period (Figure 5). We conducted this social skill training during P13–P15, which was the post-sensitive period. Social tests were performed at P13 before MI, P16 immediately after MI, and P21 for each subject (A- F). The results of the social tests were analysed using PCA (Bouquet)^33^,^34^ and summarised in a developmental trajectory, which was a quadratic approximation trajectory with calculated variance ellipses over the three social tests in each experimental group (grouped (blue), isolated (red), and ubiquinol (black)). The ellipses of Iso and Grp did not overlap on any test days. The ellipse of ubiquinol overlapped with that of Grp at P21, which was more apparent with a higher dose of ubiquinol (1200 mg/kgw·day). In a more quantitative comparison, the PCA score of each ubiquinol subject was assigned to either Grp or Iso based on the Mahalanobis distance, and the bar graph of the G ratio was computed as the subject number ratio assigned as Grp (closer to Grp than to Iso). The G ratio exceeded 0.5 at P21 (Figure 5B, C). Next, we examined ubiquinol ingestion after the sensitive period (Figure 5D). The behaviour trajectory of the ubiquinol subjects over time differed at P21 from that of Iso, but to a smaller degree than with earlier ingestion. At this point, the chicks took food pellets with or without ubiquinol that we placed inside the cage. When food was placed outside the cage (Figure 5E, F), the chicks were reluctant to take them through a cage window for the first time. Once they learned to take food placed outside, they developed social attachment in the social test even with a lower dose of ubiquinol (Figure 5F). We measured the plasma level of ubiquinol after the last social test (Figure 6c). At the higher dose (column C in Figure 6c), the plasma level of ubiquinol was significantly higher. Interestingly, the plasma level of ubiquinol at condition F (the lower dose with chicks taking food outside) was also significantly high. As a control, simply taking food outside (without ubiquinol) had no effect on either social attachment (Figure 5E) or on the plasma level of ubiquinol (Figure 6c, column E). Furthermore, the variance ellipse of each experimental condition (Figure 6b, A to F) in addition to the factor loading showed that only condition F (the low dose of ubiquinol with chicks taking food outside) was significantly correlated with the active (green) and positive (blue) parameters (Figure 6a, b, similar to Fig. 2.c). These results suggest that the experience of taking food outside improves the metabolism of ubiquinol and leads to social attachment formation.

## Discussion

In this study, we found out that a sensitive period is when a domestic chick is able to modify pre-acquired social affection. This depends on the conditions of the social interaction during the sensitive period, either the pre-acquired affection had been positive (affiliated) or negative (freeze or escaping). As the social interaction conditions, we showed a common motivation, like pecking food pellets or going-in and –out over the pen, on the while just being together without any motivated interaction leave the pre-acquired state as it was. Contrary to the proposal of a sharp critical period for the imprinting^27^, several researchers described the ending of the imprinting was not so sharp and sensitive over postnatal day 6^26^,^28^. In our experiment, the chick social behaviour was still able to change during postnatal day 5–9^39^. The difference is uncertain at this moment, but the difference of the imprinting object may be one reason. Nakamori et al^28^. used visual imprinting toward the computer-generated geometrical images^29^, whereas in this study, the real chick was used and not only visual stimuli, but also acoustic and somatosensory cues were integrated to develop social affiliation. However, there is a need to clarify precise neuronal substrate for social affiliation toward chicks before we can answer the difference of the ending of the critical period for the imprinting and the question about the nature of social affiliation learning compared with the classical imprinting.

The paper investigates the effect of synchronized behaviour based on common motivation. It could be worth mentioning the literature showing that the contingent presentation of moving stuffed-conspecific model with the test chick's distress call increase imprinting for the artificial object in Japanese quail chicks^40^. The presence of contingency in the behaviour of different agents in the chasing cognition test of infant has also a role in animacy perception in humans^41^. In addition, a couple of papers have reported some further evidence of early social predispositions and abilities demonstrated in domestic chicks^42^,^43^,^44^,^45^. Early social experience based on predisposition are hypothesised to have a role in orienting young chicks' attention toward objects that are likely to be social partners, directing the imprinting process^46^,^47^.

Another interesting finding in this study is that the experience of pecking food placed outside of the home cage was effective to acquire social affiliation and this effect was enhanced by the reduced form of CoQ10 (ubiquinol). The molecular mechanism of this phenomenon is uncertain at this moment. A plausible mechanism is the synergistic effect of energy metabolism driven by ubiquinol supplementation and social skill training based on common motivation-driven competition and corporation. Another intriguing possibility is peri-personal space modulation. Peri-personal space is a reflective defensive barrier within arm's-reach distance, on average^48^, but is largely modulated by object emotional valence^49^ and related to multi-modal attention space regulated by parieto-frontal interaction^50^ and claustrophobic fear^51^. Another possibility of the effect of synchronized behaviour is more general enhanced effect on motor and sensory activity. On the same line of the concern, the effect of ubiquinol may be general improvement of energy metabolism. The effect of generally enhanced motor, sensory activity, and improved energy metabolism, however, remains to be clarified in terms of synergistic effect of socially oriented sensory-motor activity and general physical activity. Whatever the precise mechanism is, it is reasonable to assume that the overcoming of self-boundaries with rewards (in this study, a food reward, for example) modulated the peri-personal space, improved the chicks' attention to others, and led to social attachment formation through mutual interaction via the ‘social party effect' propelled by the energy supply.

## Methods

### Animals

The animal care and handling were in accordance with institutional regulations and were approved by the animal ethics committee at the Tokyo University of Agriculture and Technology, which in turn conforms to the National Institute of Health guidelines on the care and use of laboratory animals. Fertilised eggs of white leghorn (Maria) were purchased from Tomaru Farm (Gifu) and were incubated at 37.7°C until embryonic day 20 (E20, E1 was defined as the first 24 hours after the start of the incubation). From E21, which was one day before hatching, the eggs were moved into a cage with either an isolated (Iso) or grouped (Grp) condition. The size of the Grp (basically 3–4 birds) or Iso home cages was 40w × 50d × 50h for the grouped condition or 20w × 20d × 20h for the isolation condition in centimetres. In some case, we used intermediate size 37 w × 32d × 33h for both the isolation and grouped conditions. ON/OFF light was regulated at 8am/20pm. Each home cage was surrounded with acoustic absorption materials to attenuate the call interaction among chicks in different cages. The experimental pellet for chicks (Oriental East) mixed with ubiquinol (Kaneka) or vehicle (corn oil for biochemistry, Wako) was prepared and supplied under the precise quantification control.

### Behavioural output analysis for quantification of emotional state translation (Bouquet)

Social behaviour was tested in serial contexts of meeting with unfamiliar peers. The series of contexts consisted of ‘isolation' in the test cage in the pre-set condition and then as a visual and acoustic interaction (v + a+) for two minutes in the main condition. Each subject's behaviour, which was recorded by a digital video camera (Sony or Panasonic), was analysed in serial JPEG images per second using the software TMPGEnc (Pegasys Inc., Tokyo) and through WAVE files using the free sound spectrogram software Syrinx (kindly provided by Dr. John Burt). The XY coordinate was quantified by Image J (http://rsb.info.nih.gov/ij, NIH. 29 July 2013). The correlation of the behaviour parameters based on the correlation matrix was computed using principal component analysis (PCA) software (mulvar95, http://www.vector.co.jp/soft/win95/edu/se203904.html, Kanda, K. July 29 2013 and R, http://aoki2.si.gunma-u.ac.jp/R/pca.html, and the source code was copy from http://aoki2.si.gunma-u.ac.jp/R/src/pca.R. Aoki, S. 29 July 2013). We referred to these series of behaviour analysis methods as the behavioural output analysis for quantification of emotional state translation (Bouquet)^33^,^34^. The PCA score plots of each conditional set (e.g., female or male) were represented by a regression ellipse whose long or short axis consisted of the factor loading vectors of the first and second components projected from the average centre bi-directionally with calculation by the second PCA based on the variance co-variance matrix. The factor loading vector direction and length represent the positive distribution and correlation coefficient, respectively. The length of the ellipse axes was multiplied by the square root of each eigenvalue of the first and second components. The ellipse line was coloured red or blue for Iso or Grp, respectively. 3D trajectory graphs were created using the Origin 7.5 software (Origin Lab) with quadratic curve regression drawing.

### Measurement of Ubiquinol in blood plasma

After the social tests on postnatal day 21, 200 microliters of the blood were sampled from the atrium under anaesthesia for euthanasia by isoflurane and the blood sample was centrifuged into a column tube for plasma extraction with heparin (Chatani) for 10 minutes at 2500 rpm, and the plasma was stored at −80°C. The HPLC measurement of ubiquinol was outsourced to the Kaneka Technoresearch Center within a period of three months.

### MRI analysis

The compact 2T-MRI based on a permanent magnet for small animals (DS pharmabiomedical, Suita) was used for a longitudinal volumetric scan. Each subject was scanned four times: at P4–5, P8–9, and P13 while alive and at P16 after the paraformaldhyde fixation. Each live animal was scanned with 40 phi RF coil (TR/TE 1000/100 ms, voxel size: 0.156 × 0.156 × 0.469 mm, NEX2), and its fixed brain was scanned with 30 phi RF coil (TR/TE 1800/100 ms, voxel size: 0.156 × 0.156 × 0.234 mm, NEX8). The coronal section was 3 mm thick with respect to Kuenzel, A8.8–11.0, and set at the centre position of the MRI, and the entire area of the centre section was used for the volumetric analysis. The image distortion caused by an uneven magnetic field was adjusted by referring to its 2 mm grid phantom. At P16, each animal was kept under anaesthesia for euthanasia using isoflurane. After decapitation, its head was immersed in cold 4% of paraformaldehyde (PFA) in 0.1% of phosphate buffer.

### Statistical analysis

A one-way ANOVA and Tukey's post-hoc tests were performed using Origin 7.5. Wilks' lambda (http://aoki2.si.gunma-u.ac.jp/R/wilks.html, Aoki, S. 29 July 2013) and Mahalanobis distance (http://aoki2.si.gunma-u.ac.jp/R/mahalanobis.html, Aoki, S. 29 July 2013) values were computed using the free program R.

## Author Contributions

M.K. and S.N. designed, operated and wrote the manuscript. G.K., A.S., K.M., Y.S., Y.F., S.Ob., H.S., K.I. and S.Oz. performed the experiments. T.U. and H.Y. critically read the manuscript.

## Supplementary Material

Supplementary Information Figure S1
