# Supplementary material for: Peer attachment formation by systemic redox regulation with social training after a sensitive period
Source: Sci Rep. 2013 Aug 26;3:2503. doi: 10.1038/srep02503 (PMC3752617; doi:10.1038/srep02503)
Supplement: Supplementary Information — Figure S1 [file srep02503-s1.pdf]

Supplementary Information

Title:

Peer attachment formation by systemic redox regulation with social training after a sensitive period.

Author lists:

Mamiko Koshiba, Genta Karino, Aya Senoo, Koki Mimura, Yuka Shirakawa, Soichi Fukasawa, Yuta Fukushima, Saya Obara, Hitomi Sekihara, Shimpei Ozawa, Kentaro Ikegami, Toyotoshi Ueda, Hideo Yamanouchi and Shun Nakamura

Figure S1. Visualization of social behaviour

a. Definition of seven behavioural parameters.

The coordinate of head centre was marked by a circle such as the illustration in Figure S1.1b. The circle size, the outer colour, and inner colour indicate call frequency, call type, and the object of pecking, respectively. The bar out the circle shows a beak direction.

b. Visualisation of the typical behaviour of an Iso (b1-4) or Grp subject (b5-8).

The result of the behaviour test in two contexts was presented, in the isolation context (b1-2, b5-6) and in the visual/auditory interaction (v+a+) (b3-4, b7-8). These behaviours are also illustrated in 3D in time and space by plotting the 3D coordinate (X, Y, and time). Each 3D illustration (b1, 3, 5, and 7) was paired with a plane showing local preference score (b2, 4, 6, and 8) with colour gradation depicted in Figure S1.1b9. The grouped chicks (Grp) expressed significantly higher LP-G in the v+a+ context, whereas the isolated chicks (Iso) typically showed higher LP-C in the isolation context (one-way ANOVA, n=10 each).

Figure S1

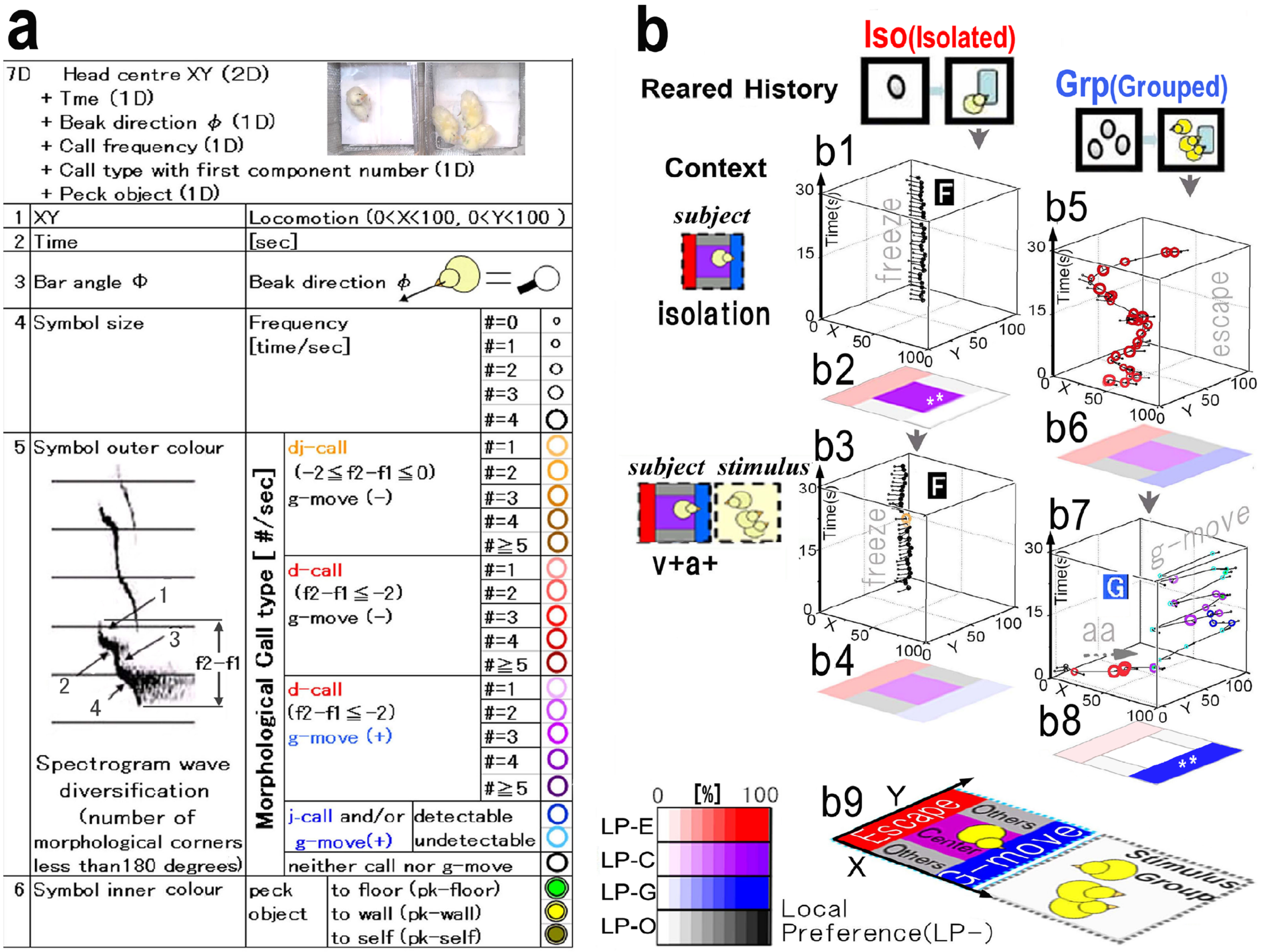

a
7D Head centre XY (2D)
+ Tme (1D)
+ Beak direction ϕ (1D)
+ Call frequency (1D)
+ Call type with first component number (1D)
+ Peck object (1D)
1 XY
Locomotion (0<X<100, 0<Y<100)
2 Time
[sec]
3 Bar angle Φ
Beak direction ϕ
4 Symbol size
Frequency [time/sec]
#=0
#=1
#=2
#=3
#=4
5 Symbol outer colour
Spectrogram wave diversification (number of morphological corners less than180 degrees)
1
2
3
4
f2−f1
Morphological Call type [#/sec]
dj−call (−2≦f2−f1≦0) g−move (−)
#=1
#=2
#=3
#=4
#≥5
d−call (f2−f1≦−2) g−move (−)
d−call (f2−f1≦−2) g−move (+)
j−call and/or g−move(+)
detectable
undetectable
neither call nor g−move
6 Symbol inner colour
peck object
to floor (pk−floor)
to wall (pk−wall)
to self (pk−self)
b
Reared History
Iso(Isolated)
Grp(Grouped)
Context
subject
isolation
subject stimulus
v+a+
b1
b2
b3
b4
b5
b6
b7
b8
b9
Time(s)
freeze
escape
g−move
aa
F
G
X
Y
LP-E
LP-C
LP-G
LP-O
0 [%] 100
Escape
Others
Center
G−move
Stimulus Group
Local Preference(LP−)
